# *trans*-Diaqua­bis­(1*H*-imidazole-4-carboxyl­ato-κ^2^ *N* ^3^,*O* ^4^)nickel(II)

**DOI:** 10.1107/S1600536811018824

**Published:** 2011-06-11

**Authors:** Shengrun Zheng, Songliang Cai, Jun Fan, Weiguang Zhang

**Affiliations:** a School of Chemistry and Environment, South China Normal University, Guangzhou 510006, People’s Republic of China

## Abstract

In the title complex, [Ni(C_4_H_3_N_2_O_2_)_2_(H_2_O)_2_], the Ni^II^ ion is located on an inversion center and shows a distorted octa­hedral geometry, defined by two *N*,*O*-bidentate 1*H*-imidazole-4-carboxyl­ate ligands in the equatorial plane and two water mol­ecules in the axial positions. Inter­molecular N—H⋯O hydrogen bonds link the complex mol­ecules into layers parallel to (10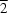), which are further linked into a three-dimensional supra­molecular network through O—H⋯O hydrogen bonds.

## Related literature

For general background to the design and synthesis of coordination polymers, see: Choi & Jeon (2003 ▶); Moulton & Zaworotko (2001 ▶); Roesky & Andruh (2003 ▶); Tao *et al.* (2000 ▶). For complexes with imidazole-4,5-dicarb­oxy­lic acid, see: Alkordi *et al.* (2009 ▶); Lu *et al.* (2009 ▶); Sun *et al.* (2005 ▶). For related structures, see: Gryz *et al.* (2007 ▶); Haggag (2005 ▶); Starosta & Leciejewicz (2006 ▶); Xu *et al.* (2008 ▶); Yin *et al.* (2009 ▶); Zheng *et al.* (2011 ▶).
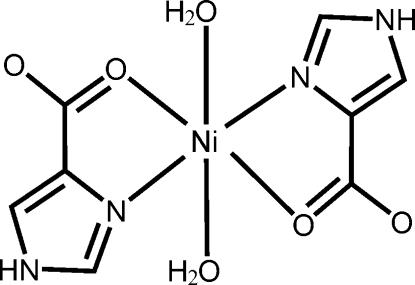


## Experimental

### 

#### Crystal data

[Ni(C_4_H_3_N_2_O_2_)_2_(H_2_O)_2_]
*M* *_r_* = 316.91
Monoclinic, 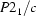

*a* = 6.6123 (18) Å
*b* = 12.267 (3) Å
*c* = 7.239 (2) Å
β = 101.059 (3)°
*V* = 576.2 (3) Å^3^
*Z* = 2
Mo *K*α radiation
μ = 1.72 mm^−1^
*T* = 298 K
0.48 × 0.36 × 0.32 mm

#### Data collection

Bruker APEXII CCD diffractometer
Absorption correction: multi-scan (*SADABS*; Sheldrick, 1996 ▶) *T*_min_ = 0.493, *T*_max_ = 0.610
2878 measured reflections
1043 independent reflections
947 reflections with *I* > 2σ(*I*)
*R*_int_ = 0.027

#### Refinement

*R*[*F* ^2^ > 2σ(*F* ^2^)] = 0.027
*wR*(*F* ^2^) = 0.070
*S* = 1.07
1043 reflections
88 parameters
H-atom parameters constrained
Δρ_max_ = 0.25 e Å^−3^
Δρ_min_ = −0.42 e Å^−3^

### 

Data collection: *APEX2* (Bruker, 2007 ▶); cell refinement: *SAINT* (Bruker, 2007 ▶); data reduction: *SAINT*; program(s) used to solve structure: *SHELXS97* (Sheldrick, 2008 ▶); program(s) used to refine structure: *SHELXL97* (Sheldrick, 2008 ▶); molecular graphics: *SHELXTL* (Sheldrick, 2008 ▶); software used to prepare material for publication: *SHELXTL*.

## Supplementary Material

Crystal structure: contains datablock(s) I, global. DOI: 10.1107/S1600536811018824/hy2425sup1.cif

Structure factors: contains datablock(s) I. DOI: 10.1107/S1600536811018824/hy2425Isup2.hkl

Additional supplementary materials:  crystallographic information; 3D view; checkCIF report

## Figures and Tables

**Table 1:** Hydrogen-bond geometry (Å, °)

*D*—H⋯*A*	*D*—H	H⋯*A*	*D*⋯*A*	*D*—H⋯*A*
N2—H2*N*⋯O2^i^	0.86	2.16	2.942 (3)	152
N2—H2*N*⋯O1^i^	0.86	2.36	3.130 (2)	149
O1*W*—H1*WA*⋯O2^ii^	0.83	1.94	2.762 (2)	169
O1*W*—H1*WB*⋯O2^iii^	0.84	1.94	2.7654 (19)	168

Symmetry codes: (i) 
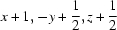
; (ii) 
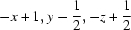
; (iii) 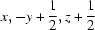.

**Fig. 1.**
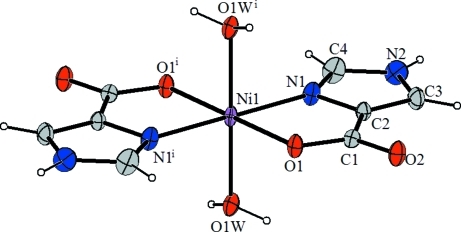
The molecular structure of the title compound, with displacement ellipsoids drawn at the 30% probability level. [Symmetry code: (i) 1 - x, -y, -z.]

**Fig. 2.**
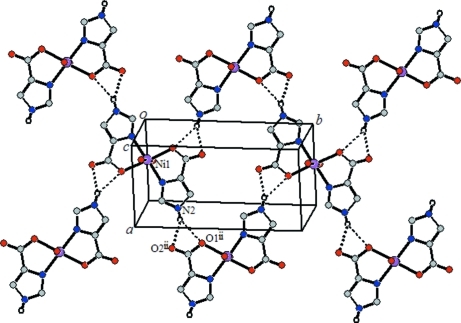
The crystal packing of the title compound, showing the two-dimensional hydrogen-bonded network. Hydrogen bonds are shown as dashed lines. [Symmetry code: (ii) x + 1, -y + 1/2, z + 1/2.]

**Fig. 3.**
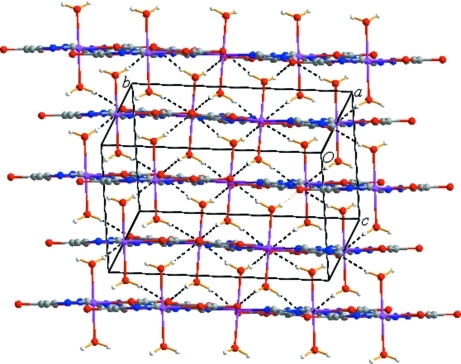
The crystal packing of the title compound, showing the three-dimensional hydrogen-bonded network. Hydrogen bonds are shown as dashed lines.

## supplementary crystallographic information

### Comment

The rational design and synthesis of coordination polymers have received extensive attention over the past decades (Moulton & Zaworotko, 2001; Roesky & Andruh, 2003). The choice of suitable ligands is an important factor that greatly affects the structure and stabilization of the coordination architecture (Choi & Jeon, 2003; Tao *et al.*, 2000). Recently, our group has focused on constructing coordination polymers based on *N*-heterocyclic carboxylic acids (Zheng *et al.*, 2011). 1*H*-Imidazole-4-carboxylic acid (H_2_imc), which is recognized as efficient N/O donors exhibiting versatile coordination behaviors and potential hydrogen-bonding abilities, remains largely unexplored, compared with its analogue imidazole-4,5-dicarboxylic acid (Alkordi *et al.*, 2009; Lu *et al.*, 2009; Sun *et al.*, 2005). A few of mononuclear complexes based on the H_2_imc ligand have been reported (Gryz *et al.*, 2007; Haggag, 2005; Starosta & Leciejewicz, 2006; Yin *et al.*, 2009). In this work, we report the synthesis and structure of a new Ni^II^ complex, which was obtained by the solvothermal reaction of Ni(ClO_4_)_2_.6H_2_O and H_2_imc.

The asymmetric unit contains a half of [Ni(Himc)_2_(H_2_O)_2_] formula unit, with the Ni^II^ ion lying on an inversion center. The Ni^II^ ion exhibits a distorted octahedral geometry, in which two bidentate chelating Himc ligands are located in the equatorial plane, forming two stable five-membered rings with metal ion, and the axial sites are occupied by two coordinated water molecules (Fig. 1). The Ni—O distances range from 2.0764 (13) to 2.0947 (17) Å and Ni—N bonds have the value of 2.0502 (17) Å, which are similar to the reported Ni^II^ complexes with imidazole-based carboxylate ligands (Xu *et al.*, 2008).

In the crystal, each complex molecule is joined to four adjacent ones *via* N2–H2···O1^ii^ and N2–H2···O2^ii^ hydrogen bonds between the imidazole N—H group and carboxylate O atoms (Table 1) [symmetry code: (ii) *x* + 1, -*y* + 1/2, *z* + 1/2], generating a two-dimensional hydrogen-bonded sheet parallel to (1 0 2) (Fig. 2). These sheets are further linked by O—H···O hydrogen bonds involving the coordinated water molecules (O1W) and carboxylate O atoms (O2), resulting in a three-dimensional supramolecular network (Fig. 3).

### Experimental

A mixture of Ni(ClO_4_)_2_.6H_2_O (41.9 mg, 0.10 mmol), H_2_imc (11.2 mg, 0.10 mmol), NaOH (4.0 mg, 0.10 mmol) and EtOH/H2O (*v*/*v* 1:1, 6 ml) was sealed in a 10 ml Teflon-lined stainless-steel reactor, which was heated to 100°C for 48 h under autogenous pressure, and then slowly cooled to room temperature at a rate of 5°C h^-1^. Pale green block crystals of the title compound were isolated, washed with distilled water and dried in air (yield: 78%). IR (KBr, cm^-1^): 3340 s, 2923 m, 2853 w, 2350 w, 1598 s, 1461 m, 1427 w, 1402 w, 1357 m, 1287 w, 1260 w, 1091 s, 1039 m, 854 w, 806 w, 723 m, 655 w, 544 m, 456w.

### Refinement

C– and N-bound H atoms were positioned geometrically and refined using a riding model, with C—H = 0.93 and N—H = 0.86 Å and with *U*_iso_(H) = 1.2*U*_eq_ (C, N). H atoms of water molecule were located from a difference Fourier map and refined as riding atoms with O—H = 0.84 Å.

### Crystal data

**Table:**

[Ni(C_4_H_3_N_2_O_2_)_2_(H_2_O)_2_]	*F*(000) = 324
*M**_r_* = 316.91	*D*_x_ = 1.826 Mg m^−^^3^
Monoclinic, *P*2_1_/*c*	Mo *K*α radiation, λ = 0.71073 Å
Hall symbol: -P 2ybc	Cell parameters from 1803 reflections
*a* = 6.6123 (18) Å	θ = 3.1–27.8°
*b* = 12.267 (3) Å	µ = 1.72 mm^−^^1^
*c* = 7.239 (2) Å	*T* = 298 K
β = 101.059 (3)°	Block, pale green
*V* = 576.2 (3) Å^3^	0.48 × 0.36 × 0.32 mm
*Z* = 2	

### Data collection

**Table:**

Bruker APEXII CCD diffractometer	1043 independent reflections
Radiation source: fine-focus sealed tube	947 reflections with *I* > 2σ(*I*)
graphite	*R*_int_ = 0.027
φ and ω scans	θ_max_ = 25.2°, θ_min_ = 3.1°
Absorption correction: multi-scan (*SADABS*; Sheldrick, 1996)	*h* = −7→7
*T*_min_ = 0.493, *T*_max_ = 0.610	*k* = −14→14
2878 measured reflections	*l* = −8→7

### Refinement

**Table:**

Refinement on *F*^2^	Primary atom site location: structure-invariant direct methods
Least-squares matrix: full	Secondary atom site location: difference Fourier map
*R*[*F*^2^ > 2σ(*F*^2^)] = 0.027	Hydrogen site location: inferred from neighbouring sites
*wR*(*F*^2^) = 0.070	H-atom parameters constrained
*S* = 1.07	*w* = 1/[σ^2^(*F*_o_^2^) + (0.0415*P*)^2^ + 0.1301*P*] where *P* = (*F*_o_^2^ + 2*F*_c_^2^)/3
1043 reflections	(Δ/σ)_max_ < 0.001
88 parameters	Δρ_max_ = 0.25 e Å^−^^3^
0 restraints	Δρ_min_ = −0.42 e Å^−^^3^

### Fractional atomic coordinates and isotropic or equivalent isotropic displacement parameters (Å^2^)

**Table:**

	*x*	*y*	*z*	*U*_iso_*/*U*_eq_	
Ni1	0.5000	0.0000	0.0000	0.02288 (16)	
N1	0.7437 (3)	0.09727 (13)	0.1157 (2)	0.0289 (4)	
C3	0.8498 (3)	0.26678 (17)	0.1929 (3)	0.0331 (5)	
H2	0.8537	0.3422	0.2070	0.040*	
C4	0.9370 (3)	0.09539 (18)	0.2052 (3)	0.0401 (6)	
H4	1.0157	0.0325	0.2312	0.048*	
C2	0.6866 (3)	0.20552 (15)	0.1070 (3)	0.0226 (4)	
N2	1.0046 (3)	0.19580 (17)	0.2536 (3)	0.0411 (5)	
H2N	1.1260	0.2125	0.3132	0.049*	
O2	0.4160 (2)	0.32925 (10)	−0.0090 (2)	0.0322 (4)	
O1	0.3604 (2)	0.15166 (10)	−0.0480 (2)	0.0270 (3)	
O1W	0.4100 (3)	−0.00996 (9)	0.2616 (2)	0.0307 (4)	
C1	0.4750 (3)	0.23191 (17)	0.0111 (2)	0.0225 (4)	
H1WA	0.4644	−0.0632	0.3236	0.027*	
H1WB	0.4254	0.0483	0.3241	0.027*	

### Atomic displacement parameters (Å^2^)

**Table:**

	*U*^11^	*U*^22^	*U*^33^	*U*^12^	*U*^13^	*U*^23^
Ni1	0.0221 (3)	0.0146 (2)	0.0284 (2)	0.00075 (12)	−0.00422 (15)	−0.00212 (12)
N1	0.0249 (10)	0.0224 (9)	0.0348 (9)	0.0037 (7)	−0.0057 (7)	−0.0029 (7)
C3	0.0268 (11)	0.0294 (11)	0.0410 (12)	−0.0075 (9)	0.0013 (9)	−0.0092 (9)
C4	0.0267 (13)	0.0401 (13)	0.0473 (13)	0.0096 (10)	−0.0089 (10)	−0.0050 (10)
C2	0.0213 (10)	0.0200 (9)	0.0249 (9)	−0.0018 (7)	−0.0001 (8)	−0.0020 (7)
N2	0.0179 (9)	0.0548 (12)	0.0455 (11)	−0.0031 (8)	−0.0066 (8)	−0.0147 (9)
O2	0.0348 (9)	0.0188 (7)	0.0385 (9)	0.0048 (6)	−0.0037 (6)	0.0020 (5)
O1	0.0211 (7)	0.0199 (7)	0.0348 (7)	0.0010 (6)	−0.0076 (6)	−0.0025 (6)
O1W	0.0403 (10)	0.0188 (7)	0.0306 (8)	0.0027 (6)	0.0008 (7)	−0.0008 (5)
C1	0.0243 (11)	0.0214 (11)	0.0204 (9)	0.0008 (7)	0.0008 (8)	0.0003 (7)

### Geometric parameters (Å, °)

**Table:**

Ni1—N1	2.0502 (17)	C4—N2	1.334 (3)
Ni1—O1	2.0764 (13)	C4—H4	0.9300
Ni1—O1W	2.0947 (17)	C2—C1	1.473 (3)
N1—C4	1.318 (3)	N2—H2N	0.8600
N1—C2	1.379 (3)	O2—C1	1.256 (2)
C3—N2	1.352 (3)	O1—C1	1.266 (2)
C3—C2	1.363 (3)	O1W—H1WA	0.83
C3—H2	0.9300	O1W—H1WB	0.84
			
N1^i^—Ni1—N1	180.00 (10)	N2—C3—H2	127.0
N1^i^—Ni1—O1^i^	80.58 (6)	C2—C3—H2	127.0
N1—Ni1—O1^i^	99.42 (6)	N1—C4—N2	110.96 (19)
N1^i^—Ni1—O1	99.42 (6)	N1—C4—H4	124.5
N1—Ni1—O1	80.58 (6)	N2—C4—H4	124.5
O1^i^—Ni1—O1	180.00 (8)	C3—C2—N1	108.92 (18)
N1^i^—Ni1—O1W^i^	90.11 (7)	C3—C2—C1	133.68 (19)
N1—Ni1—O1W^i^	89.89 (7)	N1—C2—C1	117.39 (16)
O1^i^—Ni1—O1W^i^	90.53 (5)	C4—N2—C3	108.32 (18)
O1—Ni1—O1W^i^	89.47 (5)	C4—N2—H2N	125.8
N1^i^—Ni1—O1W	89.89 (7)	C3—N2—H2N	125.8
N1—Ni1—O1W	90.11 (7)	C1—O1—Ni1	114.94 (12)
O1^i^—Ni1—O1W	89.47 (5)	Ni1—O1W—H1WA	111.4
O1—Ni1—O1W	90.53 (5)	Ni1—O1W—H1WB	114.2
O1W^i^—Ni1—O1W	180.00 (11)	H1WA—O1W—H1WB	112.4
C4—N1—C2	105.70 (17)	O2—C1—O1	123.19 (17)
C4—N1—Ni1	143.40 (15)	O2—C1—C2	120.62 (18)
C2—N1—Ni1	110.80 (13)	O1—C1—C2	116.20 (17)
N2—C3—C2	106.10 (18)		
			
O1^i^—Ni1—N1—C4	−2.4 (3)	C4—N1—C2—C1	179.45 (17)
O1—Ni1—N1—C4	177.6 (3)	Ni1—N1—C2—C1	−3.3 (2)
O1W^i^—Ni1—N1—C4	−92.9 (3)	N1—C4—N2—C3	−0.2 (3)
O1W—Ni1—N1—C4	87.1 (3)	C2—C3—N2—C4	0.2 (2)
O1^i^—Ni1—N1—C2	−177.92 (12)	N1^i^—Ni1—O1—C1	179.42 (13)
O1—Ni1—N1—C2	2.08 (12)	N1—Ni1—O1—C1	−0.58 (13)
O1W^i^—Ni1—N1—C2	91.56 (13)	O1W^i^—Ni1—O1—C1	−90.55 (13)
O1W—Ni1—N1—C2	−88.44 (13)	O1W—Ni1—O1—C1	89.45 (13)
C2—N1—C4—N2	0.2 (2)	Ni1—O1—C1—O2	178.85 (13)
Ni1—N1—C4—N2	−175.53 (18)	Ni1—O1—C1—C2	−1.03 (19)
N2—C3—C2—N1	−0.1 (2)	C3—C2—C1—O2	2.5 (3)
N2—C3—C2—C1	−179.5 (2)	N1—C2—C1—O2	−176.88 (16)
C4—N1—C2—C3	0.0 (2)	C3—C2—C1—O1	−177.7 (2)
Ni1—N1—C2—C3	177.21 (13)	N1—C2—C1—O1	3.0 (2)

Symmetry codes: (i) −*x*+1, −*y*, −*z*.

### Hydrogen-bond geometry (Å, °)

**Table:**

*D*—H···*A*	*D*—H	H···*A*	*D*···*A*	*D*—H···*A*
N2—H2N···O2^ii^	0.86	2.16	2.942 (3)	152
N2—H2N···O1^ii^	0.86	2.36	3.130 (2)	149
O1W—H1WA···O2^iii^	0.83	1.94	2.762 (2)	169
O1W—H1WB···O2^iv^	0.84	1.94	2.7654 (19)	168

Symmetry codes: (ii) *x*+1, −*y*+1/2, *z*+1/2; (iii) −*x*+1, *y*−1/2, −*z*+1/2; (iv) *x*, −*y*+1/2, *z*+1/2.
